# *PRPS*-Associated Disorders and the *Drosophila* Model of Arts Syndrome

**DOI:** 10.3390/ijms21144824

**Published:** 2020-07-08

**Authors:** Keemo Delos Santos, Eunjeong Kwon, Nam-Sung Moon

**Affiliations:** 1 Department of Biology, Developmental Biology Research Initiative, McGill University, Montreal, QC H3A 1B1, Canada; keemo.delossantos@mail.mcgill.ca; 2 Institute de Recherches Cliniques de Montréal (IRCM), Montreal, QC H2W 1R7, Canada; eunjeong.kwon@ircm.qc.ca

**Keywords:** Drosophila CRISPR, *PRPS*-associated disease, metabolic disorders, neurological disorders

## Abstract

While a plethora of genetic techniques have been developed over the past century, modifying specific sequences of the fruit fly genome has been a difficult, if not impossible task. clustered regularly interspaced short palindromic repeat (CRISPR)/Cas9 truly redefined molecular genetics and provided new tools to model human diseases in *Drosophila melanogaster*. This is particularly true for genes whose protein sequences are highly conserved. Phosphoribosyl pyrophosphate synthetase (*PRPS*) is a rate-limiting enzyme in nucleotide metabolism whose missense mutations are found in several neurological disorders, including Arts syndrome. In addition, *PRPS* is deregulated in cancer, particularly those that become resistant to cancer therapy. Notably, *Drosophila* *PRPS* shares about 90% protein sequence identity with its human orthologs, making it an ideal gene to study via CRISPR/Cas9. In this review, we will summarize recent findings on *PRPS* mutations in human diseases including cancer and on the molecular mechanisms by which *PRPS* activity is regulated. We will also discuss potential applications of *Drosophila* CRISPR/Cas9 to model *PRPS*-dependent disorders and other metabolic diseases that are associated with nucleotide metabolism.

## 1. Introduction

Inborn mutations causing metabolic disorders, particularly in purine and pyrimidine genes, often result in a variety of nervous system disorders [1,2,3,4]. However, the pathogenesis of their neurological anomalies is not well-understood, largely due to the extreme rarity of these diseases. While model organisms, like mice, could provide important insights into mechanisms of pathogenesis and treatment options, one has to consider the cost-benefit analysis of various approaches. For these reasons, *Drosophila melanogaster* is an excellent system to model rare diseases affecting the nervous system. These animals are relatively inexpensive and studies over the past decades demonstrated the validity of *Drosophila* as a model organism of neurological disorders [5,6,7,8,9]. Importantly, with the advent of clustered regularly interspaced short palindromic repeat (CRISPR)/Cas9 gene editing, patient-derived missense mutations can now be directly recapitulated in *Drosophila*. Obviously, the suitability of such an approach would depend on the degree of sequence and functional conservation between the human and *Drosophila* orthologs. One such metabolic gene is phosphoribosyl pyrophosphate synthetase (*PRPS*), the rate-limiting enzyme in nucleotide metabolism. The *Drosophila* *PRPS* ortholog is highly conserved to its human counterparts, showing about 90% protein sequence identity [10]. Among the three human orthologs, missense mutations of *PRPS1* are found in a number of rare neurological disorders such as Arts syndrome. More recently, studies have also discovered that *PRPS1* and *PRPS2* are mutated in therapy-resistant relapsed ALL cancer patients. In this review, we will summarize the role of *PRPS* mutations in human diseases, including cancer, and describe recent studies revealing the molecular mechanisms by which *PRPS* activity is regulated. We will then describe how *Drosophila* models helped to uncover unexpected physiological consequences of *PRPS* mutations and discuss the potential application of CRISPR/Cas9 genome editing in *Drosophila*.

## 2. Phosphoribosyl Pyrophosphate Synthetase (*PRPS*) Is a Rate-Limiting Enzyme in Nucleotide Metabolism

Nucleotides, composed of a nitrogenous base, a five-carbon sugar ribose, and a phosphate group, are essential for a myriad of fundamental molecular and cellular processes. For example, purine and pyrimidine nucleotides are the building blocks of DNA and RNA. Likewise, pyridine nucleotides are important co-factors for many important biochemical reactions. *PRPS* is the rate-limiting enzyme in nucleotide biosynthesis that is responsible for the transfer of the β-γ diphosphate of ATP to the C1 hydroxyl of ribose-5-phosphate (R5P), which is a compound produced by the pentose phosphate pathway [11]. This produces phosphoribosyl pyrophosphate (PRPP), a common precursor of the five-carbon sugar unit of all nucleotides. The following section briefly describes how PRPP is used during purine and pyrimidine nucleotide biogenesis. 

De novo synthesis of purine nucleotides involves the assembly of the purine moiety directly onto PRPP in a 10-step reaction to form inosine monophosphate (IMP) [11]. IMP is then used to synthesize either adenosine monophosphate (AMP) or guanosine monophosphate (GMP). Purine nucleotides can also be synthesized through a less energy-demanding salvage pathway in which purine bases, adenine and guanine, are recycled to from AMP and GMP via phosphoribosyltransferases in a PRPP-dependent reaction. Similar to its role in purine nucleotide biosynthesis, PRPP is at the center of de novo pyrimidine nucleotide production. However, unlike purines, the pyrimidine ring is first synthesized with PRPP at a later step to produce uridine-5′-triphosphate (UTP) and orotidine monophosphate (OMP) de novo [12]. PRPP is also used in the pyrimidine salvage pathway in a similar manner as the purine salvage pathway. Notably, many metabolic enzymes involved in purine and pyrimidine metabolism are mutated in rare genetic disorders, and the inborn mutations affecting those enzymes can either activate or decrease the enzyme function [3,4]. In summary, *PRPS* is the rate-limiting enzyme in nucleotide metabolism as it produces PRPP, a critical molecule for nucleotide biogenesis.

## 3. *PRPS* Is Highly Conserved 

Consistent with its essential role in nucleotide synthesis, *PRPS* is highly conserved across all domains of life (Figure S1 and [11]). There exists multiple *PRPS* orthologs among many eukaryotic organisms. Interestingly, while some yeast and plant species contain up to five *PRPS* orthologs, humans and rats have only three *PRPS* genes [11]. Both human *PRPS1* and *PRPS2*, located on the X chromosome, are expressed in all tissues, while *PRPS3* (PRPS1L1), located on chromosome 7, is restricted to the testes [13]. The structure of human *PRPS1* has been solved and shown to be similar to *PRPS* from *Bacillus subtilis* (*B. subtili*) despite limited sequence identity [14]. *PRPS* from both species form hexamers that are comprised of three homodimers arranged in a propeller-shaped tertiary structure. Interestingly, in addition to forming hexametric structures, a recent study has demonstrated that *PRPS* can form cytophilic filaments [15,16]. These are evolutionarily conserved intracellular structures, sometimes referred to as cytophidia, which are thought to increase enzyme kinetics in response to various stressors (e.g., RNA stress granules, nutrient deprivation). Such structures are observed with other metabolic enzymes such as CTP synthase [16,17]. Strikingly, *PRPS* cytophilic filament formation is also conserved in a wide range of species including bacteria, zebrafish, rats, and humans, illustrating the functional importance of *PRPS* in all living species [16]. In humans, there are also *PRPS*-associated proteins (PAPs), PAP39 and PAP41, which share some sequence homology with *PRPS* (Figure S1 and [11]). PAPs were shown to physically interact with *PRPS*. However, PAPs lack a catalytic ATP binding site and do not directly participate in the phosphoribosyltransferase reaction but rather play a regulatory function [11,18]. 

*Drosophila* markedly has only one *PRPS* ortholog (*dPRPS*), coded by *CG6767*, which was recently characterized [10] and shares about 90% protein sequence identity with human *PRPS1* [10]. Based on the high sequence similarity, the structure of *dPRPS* must resemble that of the human counterpart. Indeed, *dPRPS* cytophilic filament formation has been also observed in the *Drosophila* ovary [16]. In addition, *Drosophila* contains a single PAP ortholog *CG2246*, which is yet to be characterized. Although not as highly conserved as *PRPS*, *CG2246* encodes a protein that is 57% and 63% identical to PAP39 and PAP41, respectively. The sequence similarity and the presence of the PAP ortholog make *Drosophila* an attractive genetic system to study the in vivo function of *PRPS*. Moreover, the fact that *dPRPS* and *CG2246* are the only orthologs in *Drosophila* eliminates any issues of functional redundancy between family members, making genetic studies uncomplicated and straightforward.

## 4. *PRPS* Mutations in Neurological Disorder

Inborn mutations of *PRPS1* are associated with a number of neurological disorders with a broad range of pathological symptoms [1,2]. This is not surprising given the ubiquitous nature of purine, pyrimidine, and pyridine nucleotides in basic molecular processes as fundamental as synthesizing DNA and RNA. At the same time, it is truly difficult to determine which metabolic pathway downstream of *PRPS* is responsible for the pathology of *PRPS1*-associated neurological disorders. In fact, inborn mutations in many other purine and pyrimidine genes downstream of *PRPS1* also result in a variety of nervous system disorders [3,4]. Interestingly, *PRPS1* mutations associated with neurological disorders can be either a loss-of-function or gain-of-function mutation. Regardless of their effect on enzymatic activity, a common symptom associated with most *PRPS1* mutations is hearing impairment (either nonsyndromic or syndromic hearing loss). For some reason, sensory neurons in the auditory system appear highly susceptible to defects in nucleotide metabolism. The 29 *PRPS1* mutations identified in neurological disorders are summarized in Table 1. Notably, approximately three-quarters are associated with loss-of-function mutations in X-linked neurological disorders. Gain-of-function mutations of this enzyme are associated with *PRPS* superactivity, with eight missense mutations reported thus far. Interestingly, a recent study identified a mutation, V142L, that may have both positive and negative effects on enzyme function, as it may affect the allosteric site as well as the ATP-binding site [19]. Whether the mutations in patients increase or decrease *PRPS1* function, there is a paucity of treatment options and they only aim to slow disease progression and are not curative [20].

Interestingly, every *PRPS1* mutation, both hypoactivating and hyperactivating *PRPS1* mutations, are all missense mutations and, thus far, no nonsense mutation has been identified in the patients. This likely reflects the essential nature of *PRPS1* in nucleotide metabolism for embryonic development and suggests that neither *PRPS2* nor *PRPS3* can compensate for *PRPS1* function. This also indicates that the mutant form of *PRPS1* in patients must retain some activity that allows affected individuals to complete embryonic and fetal development. Supporting this notion, structural analysis of mutant *PRPS1* has revealed that, while most nonsense mutations affect either substrate binding, dimer interface, or secondary structure, none of them disrupt the overall structure of *PRPS1* [21]. The spectrum of X-linked neurological disorders that are associated with decreased *PRPS1* function includes Arts syndrome, Charcot-Marie-Tooth disease 5 (CMTX5), nonsyndromic sensorineural deafness 2 (DFN2), and more recently retinal dystrophy [22,23,24]. Notably, the severity of symptoms associated with these disorders likely reflects the extent to which the enzyme function is disrupted. Arts syndrome is characterized by the most severe disease presentation, such as mental retardation, early-onset hypotonia, ataxia, profound congenital sensorineural hearing impairment, and optic atrophy, as well as early-childhood death [23]. There have been excellent reviews delineating these disease phenotypes and will not be further discussed in this review [20,25]. *PRPS1* overactivity is caused by missense mutations in the allosteric site that lead to reduced inhibition by its allosteric inhibitor, ADP, and increased stimulation by phosphate [25]. The physiological consequences of *PRPS1* overactivity include neurosensory defects, hyperuricemia, and gout, caused by an accumulation of uric acid, which is the end-product of purine catabolism. Gain-of-function mutations were first discovered in patients with gout presenting with high levels of uric acid [26]. 

Because *PRPS1* is an X-linked gene, *PRPS1*-associated neurological disorders, caused by either loss-of-function or gain-of-function mutations, primarily affect males. Although less susceptible and frequent, females can also present with symptoms of *PRPS1*-associated disorders even if they are heterozygous for the mutation. A recent study found a female patient bearing a novel *PRPS1* hyperactivating mutation [27]. Although both the patient and her mother carried a single copy of the mutant allele, the patient presented with symptoms associated with *PRPS1* superactivity while her mother was largely unaffected. This finding demonstrated that heterozygous females can be affected by *PRPS1* mutation if a skewed X-inactivation occurs during development. A similar observation was also made with *PRPS1* loss-of-function mutations. Novel *PRPS1* missense mutations were identified to cause retinal dystrophy in female patients, who were all heterozygous for the mutant alleles [28]. These findings also suggest that *PRPS1*-associated genetic disorders may be more prevalent than previously thought. Lastly, loss-of-function mutations in *PRPS1* have been identified in cell lines of Hutchinson–Gilford progeria syndrome (HGPS), a rare disease characterized by accelerated aging [29]. This raises the possibility that *PRPS1*-associated disorders may not be limited to neurological disorders. Overall, *PRPS1* mutations identified from human patients support the notion that *PRPS1* is a critical enzyme required for nucleotide metabolism.

## 5. *PRPS* in Cancer

Recent studies have identified the role of *PRPS* in cancer, particularly in the context of gaining drug resistance to thiopurine and thioguanine cancer therapy in childhood acute lymphoblastic leukemia (ALL) (Table 2 and [30,31]). The *PRPS1* mutation in ALL was first identified by whole exome-sequencing of samples from patients with pediatric relapsed ALL treated with thiopurine. Thiopurine is a key component of ALL chemotherapy, a prodrug that is converted by the purine salvage pathway to become a cytotoxic compound. It is proposed that cancer cells gain resistance to thiopurine by acquiring hyperactivating *PRPS1* mutations that bypass ADP- or GDP-dependent allosteric inhibition. These mutations make cancer cells favor de novo nucleotide synthesis and become less sensitive to the drugs whose efficacy depends on the purine salvage pathway [30]. Interestingly, a study also showed that cell lines with a relapse-specific *PRPS1* mutation become hypersensitive to 5-fluorouracil (5-FU), an antimetabolite drug that targets de novo pyrimidine biosynthesis [32]. This study supports the notion that the growth and survival of thiopurine-resistant cancer cells largely depend on de novo nucleotide biosynthesis. More recently, a large-scale whole-genome sequencing was performed to identify therapy-resistant specific mutations in relapsed ALL patients [31]. This study identified hyperactivating mutations in not only *PRPS1*, but also in *PRPS2*, demonstrating the crucial role of *PRPS* in nucleotide metabolism. The role of *PRPS* in ALL is further demonstrated by the observation that *PRPS1* overexpression increases proliferation and inhibits apoptosis in B-ALL cell lines [33]. Of note, many of the hyperactivating *PRPS1* mutations found in relapsed ALL occur in the same amino acid residues mutated in *PRPS1* superactivity listed in Table 1.

In addition to its role in chemotherapy resistance in ALL, increased *PRPS* function may generally promote tumorigenesis. Indeed, gain-of-function mutations have also been identified in colorectal cancer and breast cancer [34]. Moreover, analysis using publicly available resources also supports this notion. The Catalogue Of Somatic Mutations In Cancer (http://cancer.sanger.ac.uk/cosmic) shows that *PRPS1* is overexpressed in a number of cancers, such as lung squamous cell carcinoma, lung adenocarcinoma and colorectal adenocarcinoma. Furthermore, cBioPortal for Cancer Genomics (https://www.cbioportal.org/) indicates that all three *PRPS* orthologs are amplified in bladder/urinary tract and prostate cancers. Overall, recent findings clearly demonstrated the crucial role of *PRPS* in the proliferation and survival of cancer cells.

## 6. Post-Translational Modification of *PRPS*

In the past few years, several studies have identified post-translational modifications that regulate *PRPS* activity. Ketohexokinase (KHK) is a key enzyme that regulates fructose metabolism. KHK phosphorylates fructose into fructose-1-phosphate, which is then metabolized to dihydroxyacetone phosphate and glyceraldehyde-3-phosphate, two intermediate metabolites of glycolysis. Interestingly, there are two alternatively spliced forms of KHK, KHK-A, and KHK-C. While KHK-C efficiently phosphorylates fructose, KHK-A cannot. Surprisingly, KHK-A is a protein kinase that phosphorylates *PRPS1* at Thr225, which increases *PRPS1* function [35]. In hepatocellular carcinoma, it has been demonstrated that c-Myc-promotes expression of KHK-A over KHK-C and coordinately decreases fructose catabolism and increases de novo nucleotide synthesis. Importantly, KHK-dependent *PRPS1* phosphorylation is shown to be a predictor of poor prognosis in hepatocellular cancers. Another kinase that couples oncogenic changes to *PRPS1* function is cyclin-dependent kinase 1 (CDK1) [36]. CDK1-dependent phosphorylation at Ser103 activates *PRPS1* and couples the cell cycle to nucleotide metabolism. Ser103 phosphorylation of *PRPS1* is suggested to be required for maintaining increased *PRPS1* activity in colorectal cancer cells. Notably, Ser103 is one of the amino acids that is frequently mutated in relapsed ALL patients, although they are not phosphomimetic mutations (Table 2). A recent study also found a phosphorylation event that inactivates *PRPS*. Upon glucose starvation, AMP-activated kinase (AMPK) phosphorylates Ser180 of *PRPS1* and Ser183 of *PRPS2* [37]. Unlike phosphorylation by KHK-A and CDK1, AMPK-dependent phosphorylation was shown to inhibit *PRPS1/2* by preventing hexamer formation. This limits nucleotide synthesis under stressed conditions like nutrient deprivation. Apart from phosphorylation, *PRPS2* has been shown to be arginylated by arginyltransferase ATE1 [38]. While ATE1 selectively arginylates and stabilizes *PRPS2* and not *PRPS1*, the biological context in which the arginylation regulates nucleotide metabolism is yet to be determined. Overall, these findings highlight post-translational modifications of *PRPS* which couple nucleotide metabolism to various cellular signals and processes. 

## 7. Model Systems of *PRPS*-Associated Disorder

### 7.1. Mouse Models of PRPS-Associated Disorders

Although knockout mice of *PRPS1* and *PRPS2* have been generated, neither were designed to interrogate *PRPS*-associated neurological disorders. *PRPS1*-knockout mice were generated as a part of a high-throughput screening searching for mouse genes important for skeletal phenotypes [39]. Like human *PRPS1*, murine *PRPS1* is on the X chromosome and the study identified it as an X-linked gene that is required for animal viability and no further analysis was performed. *PRPS2*-knockout mice were generated in the context of a Myc-driven cancer model. In contrast to *PRPS1*-knockout mice, *PRPS2*-knockout mice are viable and fertile with no discernable developmental defects [40]. It is probable that *PRPS1* mainly contributes to overall *PRPS* activity during development and that *PRPS1* can compensate for the loss of *PRPS2*. Interestingly, while *PRPS2* is nonessential for development, it was required for *Myc*-dependent cancer formation. Not only is *PRPS2* expression regulated by Myc, but *PRPS2*-knockout mice are also resistant to *Eµ–Myc*-driven cancer development. This study suggests that at least in mice, *PRPS2* is specifically targeted by Myc to promote tumorigenesis. Notably, neither of the *PRPS*-knockout mice were analyzed for any evidence of neurological symptoms. Next, we will summarize zebrafish and *Drosophila* models that were designed to investigate *PRPS*-associated neurological diseases.

### 7.2. Zebrafish Models of PRPS-Associated Disorders

There are two *PRPS* paralogs in zebrafish, *prps1a* and *prps1b*. A *prps1a* mutant was generated by retroviral vector insertion and *prps1b* was targeted by a zinc finger nuclease, creating a frameshift mutation in exon 2 [41]. Genetic studies using these mutants revealed that *prps1a* plays a predominant role during development. While *prps1a* showed defects in the eye and the neuromast hair cells, the *prps1b* mutant displayed no discernable phenotype. However, *prps1a;prps1b* double mutants presented with more severe defects in the eye and the neuromast hair, indicating that *prps1b* does contribute to overall *PRPS* function during development. Importantly, *prps1a;prps1b* double mutants showed many features that are associated with *PRPS1*-associated neurological disorders such as defects in inner ear hair cells and motor neurons. A later study using morpholino oligonucleotides targeting *prps1a* and *prps1b* further validated zebrafish as a disease model, as they showed similar defects including significant sensorineural hearing loss [42]. One of the striking findings in the zebrafish studies is that many defects observed in *prps1a and prps1a;prps1b* mutants ameliorate with time. The authors proposed that this could be due to the fundamental difference in tissue development and homeostasis between zebrafish and humans. Interestingly, the study also revealed that while PRPP levels were severely reduced in *prps1a;prps1b* mutants, the levels of purine nucleotides, IMP, GTP, and AMP, were only reduced by about 25%. In fact, the levels of s-adenosylmethionine (SAM), a purine metabolite, were increased in *prps1a;prps1b* mutants. Perhaps, there is a metabolic reprogramming event in *PRPS*-deficient animals, which may contribute to their survival. Nevertheless, these studies have demonstrated that zebrafish are an excellent model of *PRPS1*-associated disorders, recapitulating many symptoms presented by the patients.

### 7.3. Drosophila Models of PRPS-Associated Disorders

Ever since it was first adapted for the use of genome engineering in *Drosophila* [43], the CRISPR/Cas9 system has been routinely used to generate precise and efficient edits to the fly genome. Moreover, the development of additional CRISPR-based tools to probe and interrogate the fly genome has been rapidly expanding in the *Drosophila* field. For example, with the combination of already existing tool kits such as the GAL4-UAS binary system, *Drosophila* geneticists can now carry out CRISPR/Cas9-mediated gene disruption in a tissue-specific manner. Moreover, libraries containing guide RNA (gRNA)-expressing plasmids and fly lines are being generated, which will allow genetic screening via the CRISPR/Cas9 system [44,45]. Notably, variant forms of Cas9 nucleases are also used in *Drosophila* that allow overexpression of a gene of interest from its endogenous promoter [46] These techniques will further improve *Drosophila* as a model organism to probe and interrogate gene function.

In our group, we used CRISPR/Cas9 techniques to engineer two *Drosophila* alleles carrying equivalent mutations identified from Arts syndrome [10]. While null alleles were generated in other model organisms, the physiological consequence of disease-causing *PRPS1* mutations has not been directly tested. We reasoned this was important since having no *PRPS1* versus severely reduced *PRPS1* may have different physiological consequences. Two mutant alleles carrying *PRPS1* mutations identified from Arts syndrome, Q133P and R196W, were engineered in flies (Table 1). Initially, we were surprised by the fact that the two *dPRPS* alleles are viable and fertile with no discernable development defects, given its essential role in nucleotide metabolism. However, this also supported the notion that *PRPS1* missense mutations from patients do not completely eliminate enzyme function. In fact, publicly available insertion mutant alleles of *dPRPS*, which are transcript null, are early larval lethal. One of the advantages of the *Drosophila* system is the ability to systematically examine a vast range of phenotypes ranging from cellular to behavioral defects owing to more than 100 years of research history. Despite the lack of morphological defects, we were able to quickly learn that *dPRPS1* alleles carrying patient-derived mutations have clear signs of neurodegeneration. The adult flies had much shorter lifespans than controls and displayed locomotor defects, which are measured by a climbing assay [47]. In addition, using various molecular genetic tools, we were able to determine that mutant flies have profound defects in lipid mobilization, macroautophagy, and lysosome function, all of which are known to be associated with neurological disorders [10]. In fact, we were able to demonstrate that adult brains of *dPRPS* mutants accumulate ubiquitinated proteins suggesting that macroautophagy/lysosomal defects affect the nervous system. These cellular defects led to the discovery that, in addition to aging and locomotor defects, *dPRPS*-mutant flies are also hypersensitive to starvation and oxidative stress. Importantly, it has not yet been investigated whether patients with *PRPS1* mutations have autophagy/lysosomal defects nor are hypersensitive to starvation and oxidative stress. However, these *Drosophila* models will likely prove to be valuable in understanding the mechanism underlying *PRPS1*-related disorders. A clinical trial showed that therapeutic SAM treatment delays the onset of neurodegeneration in Arts syndrome patients [2]. Validating *dPRPS* mutant flies as a model of Arts syndrome, dietary supplementation of SAM can partially improve lysosome dysfunction and sensitivity to starvation in *dPRPS*-mutant flies [10]. Collectively, by engineering *Drosophila* carrying patient-derived mutations, we uncovered that patient-derived *PRPS* mutations are likely hypomorphic, maintaining a certain level of enzyme activity, and that cellular defects such as macroautophagy/lysosomal defects likely contribute to *PRPS*-associated neuropathology. Furthermore, these *Drosophila* models of Art syndrome could potentially be used to test the efficacy of future treatments.

## 8. Investigating Inborn Error in Metabolism Using *Drosophila* CRISPR/Cas9

While different methods exist to model disease-associated mutations in *Drosophila*, several factors made *PRPS1* mutations ideal candidates to model disease in *Drosophila* via CRISPR/Cas9. First, the *dPRPS* protein sequence is highly conserved from human *PRPS1*. This high conservation combined with the available structural data made certain that the patient-derived missense mutations would have the same molecular effects on the *Drosophila* *PRPS*. Second, every single *PRPS1* inborn mutation identified from patients was a missense mutation and that the in vivo consequences of these mutations have not directly been studied. Third, there is only one *PRPS* ortholog in *Drosophila*, which shows high sequence and protein homology to human *PRPS1*. These three factors made the analysis and interpretation of the data in patient-derived *Drosophila* mutants straightforward. 

A plethora of missense mutations associated with an inborn error in purine and pyrimidine metabolisms has been identified. Similar to *PRPS1* mutations, different mutations in a single gene may result in a wide range of disease phenotypes [3,4]. With relatively low-cost and short generation time, *Drosophila* can be used to potentially model every disease-causing mutation. However, the practicality of this approach is dependent on the conservation between humans and *Drosophila*. For instance, our sequence analysis of the human and *Drosophila* orthologs of few genes in purine metabolism reveals limitations to this approach. Table 3 lists selected genes in purine metabolism whose deficiencies are associated with rare metabolic disorders [3]. ADSL (adenylosuccinate lyase) and ATIC (5-aminoimidazole-4-carboxamide ribonucleotide formyltransferase/IMP cyclohydrolase) are critical enzymes in de novo purine biosynthetic pathways and their deficiencies have been associated with a number of neurological features [3]. Similar to *PRPS1*, both *ADLS* and *ATIC* have clear *Drosophila* orthologs whose protein sequence shows a high degree of similarity, 78.7% and 83.3% respectively, making them ideal candidates. Interestingly, both enzymes are components of the purinosome, a multiprotein complex consisting of enzymes that carry out de novo purine biosynthesis [48]. Perhaps, the need to form a complex quaternary structure, similar to hexamer formation of *PRPS*, puts evolutionary pressure to conserve protein sequences among these enzymes. *PNP* (purine nucleoside phosphorylase), *ADA* (adenosine deaminase), *APRT* (adenine phosphoribosyltransferase) and *HPRT1* (hypoxanthine phosphoribosyltransferase 1) are involved in purine catabolism and salvage pathway and their *Drosophila* orthologs have varying degrees of sequence similarity. *PNP* and *APRT* do have clear *Drosophila* orthologs but show relatively low sequence similarities, 55.6% and 62.7%, respectively. For such genes, the nature and location of the mutations from patients would determine whether such mutations are suitable to model in *Drosophila* via CRISPR/Cas9. If missense mutations are located in a well-conserved region and structural information of the enzymes are available, *Drosophila* mutants carrying patient-derived mutations would be informative. Surprisingly, *HPRT1* (hypoxanthine phosphoribosyltransferase 1) and *ADA* (adenosine deaminase), whose deficiency is associated with Lesch–Nyhan disease and SCID (Severe combined immunodeficiency) respectively, have no clear sequence orthologs in *Drosophila*. This is striking given the crucial function that HPRT1 and ADA play in purine salvage and catabolic pathway. Perhaps other *Drosophila* proteins with similar enzymatic activities carry out their functions. It is also possible that there is a fundamental difference between human and *Drosophila* purine metabolism that is yet to be discovered. Nevertheless, it would be difficult to use *Drosophila* to investigate inborn errors of such genes. In summary, the extent of sequence similarity, structural information, and mechanistic data will determine whether *Drosophila* is a suitable system to model a disease-associated mutation. 

## 9. Concluding Remarks

With sophisticated genetic tools such as the Gal4/UAS binary system and the FRT/FLP recombination system, *Drosophila* has thrived for many years as a simple yet elegant genetic system [49]. However, an inability to modify the genome in a sequence-specific manner had been the Achilles heel for *Drosophila* geneticists and other model systems such as yeast and mice had advantages over the fruit fly in this respect. The introduction of CRISPR/Cas9 in the past few years has leveled the playing field and changed the way *Drosophila* scientists plan and design their experiments. For example, in the future, we can engineer *dPRPS* alleles carrying patient-derived gain-of-function mutations or directly determine the physiological significance of the *PRPS* phosphorylation sites described above. With improved sequencing technology, an increasing number of disease-associated mutations are being identified. In addition, novel CRISPR/Cas9 technology is constantly being developed, thus enhancing our ability to edit the genome. For example, a modified Cas9 endonuclease fused with a reverse transcriptase has recently been engineered [50]. This variant form of Cas9 together with a modified guide RNA allows precise genome editing without introducing double-stranded breaks on DNA nor requiring any donor template DNA. Overall, CRISPR-based techniques together with already existing elegant genetic tools make *Drosophila* an attractive organism to study and interrogate the effect and mechanisms underlying patient-derived mutations.

## Figures and Tables

**Table 1:** Missense mutations identified in phosphoribosyl pyrophosphate synthetase 1 (*PRPS1*) associated with *PRPS1*-associated neurological disorders.

Disorder	Effect on *PRPS1* Function	Mutation	Amino Acid Change
*PRPS-1* Superactivity	Gain of Function	154G > C [51]	D52H
		341A > G [52]	N114S
		385C > A [51]	L129I
		521G > T [27]	G174V
		547G > C [52]	D183H ^1^
		569C > T [53]	A190V ^1^
		578A > T [54]	H192L
		579C > G [51]	H193Q ^1^
Nonsyndromic X-linked sensorineural deafness (DFN2)	Loss of Function	193G > A [55]	D65N
		259G > A [55]	A87T
		869T > C [55]	I290T
		916G > A [55]	G306R
Charcot-Marie-Tooth neuropathy type 5 (CMTX5)	Loss of Function	129A > C [56]	E43D
		334G > C [24]	V112L
		344T > C [56]	M115T
Arts syndrome	Loss of Function	398A > C [23]	Q133P
		455T > C [23]	L152P
		856C > T [22]	R196W
*PRPS-1* Superactivity and Arts syndrome	Loss of Function	424G > C [19]	V142L
CMTX5 and Arts syndrome	Loss of Function	830A > C [57]	Q277P
DFN2 and CMTX5	Loss of Function	337G > T [58]	All3S
DFN2 and CMTX6	Loss of Function	343A > G [58]	M115V
DFN2 and CMTX7	Loss of Function	925G > T [58]	V309F
DFN2 and CMTX8	Loss of Function	62C > G [59]	A121G
Retinal Dystrophy	Loss of Function	46T > C [60]	S16P
		47C > T [28]	S16F
		586C > T [28]	R196W
		640C > T [28]	R214W
		641G > C [28]	R214P

Amino acid number adjusted based on UNIPROT data.

**Table 2:** Missense mutations of *PRPS* identified in human cancer.

Cancer Type	Gene	Amino Acid Change
Relapse-specific ALL	*PRPS1*	V53A [30]
		I72V [30]
		C77S [30]
		S103I [30]
		S103N [30]
		S103T [30]
		S103R [31]
		N114D [30]
		D139G [30]
		N144S [30]
		G174E [30]
		K176N [30]
		R177S [31]
		D183E [30]
		A190V [30]
		A190T [30]
		L191F [30]
		T303S [30]
		Y311C [30]
		V316L [31]
	*PRPS2*	V48M [31]
		S120S [31]
		A134T [31]
		P173Y [31]
		A175T [31]
Breast Cancer	*PRPS1*	D203H [34]
		V219G [34]
Colorectal Cancer	*PRPS1*	H231D [34]

**Table 3:** Genes causing inborn errors in purine metabolism.

Gene	ensID	Gene Name	Drosophila Homologues	FB ID	Homology between Human and Fruit Fly Orthologs
*ADSL*	ENSG00000239900	adenylosuccinate lyase (*de Novo*)	***AdSL***	FBgn0038467	Identity: 65.6%
					Similarity: 78.7%
*ATIC*	ENSG00000138363	5-aminoimidazole-4-carboxamide ribonucleotide formyltransferase/IMP cyclohydrolase (*de Novo*)	***CG11089***	FBgn0039241	Identity: 70.6%
					Similarity: 83.3%
*PNP*	ENSG00000198805	purine nucleoside phosphorylase (Catabolism)	***CG16758***	FBgn0035348	Identity: 44.6%
					Similarity: 59.8%
*ADA*	ENSG00000196839	adenosine deaminase (Catabolism)	***ADA (?)***	FBgn0037661	Identity: 23.7%
					Similarity: 38.8%
*APRT*	ENSG00000198931	adenine phosphoribosyltransferase (Salvage)	***APRT***	FBgn0000109	Identity: 44.3%
					Similarity: 62.7%
*HPRT1*	ENSG00000165704	hypoxanthine phosphoribosyltransferase 1 (Salvage)	**Unknown**	-	-

Selected genes associated with inborn error in purine metabolism and their Drosophila orthologs are shown. Protein sequence homology is determined by an alignment tool from EMBL-EBI (www.ebi.ac.uk/Tools/psa/).

## Supplementary Materials

The following are available online at https://www.mdpi.com/1422-0067/21/14/4824/s1, Figure S1: Phylogenetic tree of *PRPS* and *PRPS*-binding proteins (PAP) across species. Phylogenetic relationships of *PRPS* homologs across species were estimated using PhylomeDB (http://phylomedb.org/). Red indicates duplication events. Blue indicates speciation events. Green dot marks the target sequence, human *PRPS1*. Q9VT33 is the only Drosophila *PRPS* ortholog. *PRPS*-associated protein 1 and 2 (PRPPSAP1 and PRPPSAP2) are orthologues of human PAP39 and PAP41, respectively. Conserved domains of *PRPS*-family are shown in the right panel.
