# Supplementary material for: PRPS-Associated Disorders and the Drosophila Model of Arts Syndrome
Source: Int J Mol Sci. 2020 Jul 8;21(14):4824. doi: 10.3390/ijms21144824 (PMC7403961; doi:10.3390/ijms21144824)
Supplement: Supplementary file 1 [file ijms-21-04824-s001.docx]

**
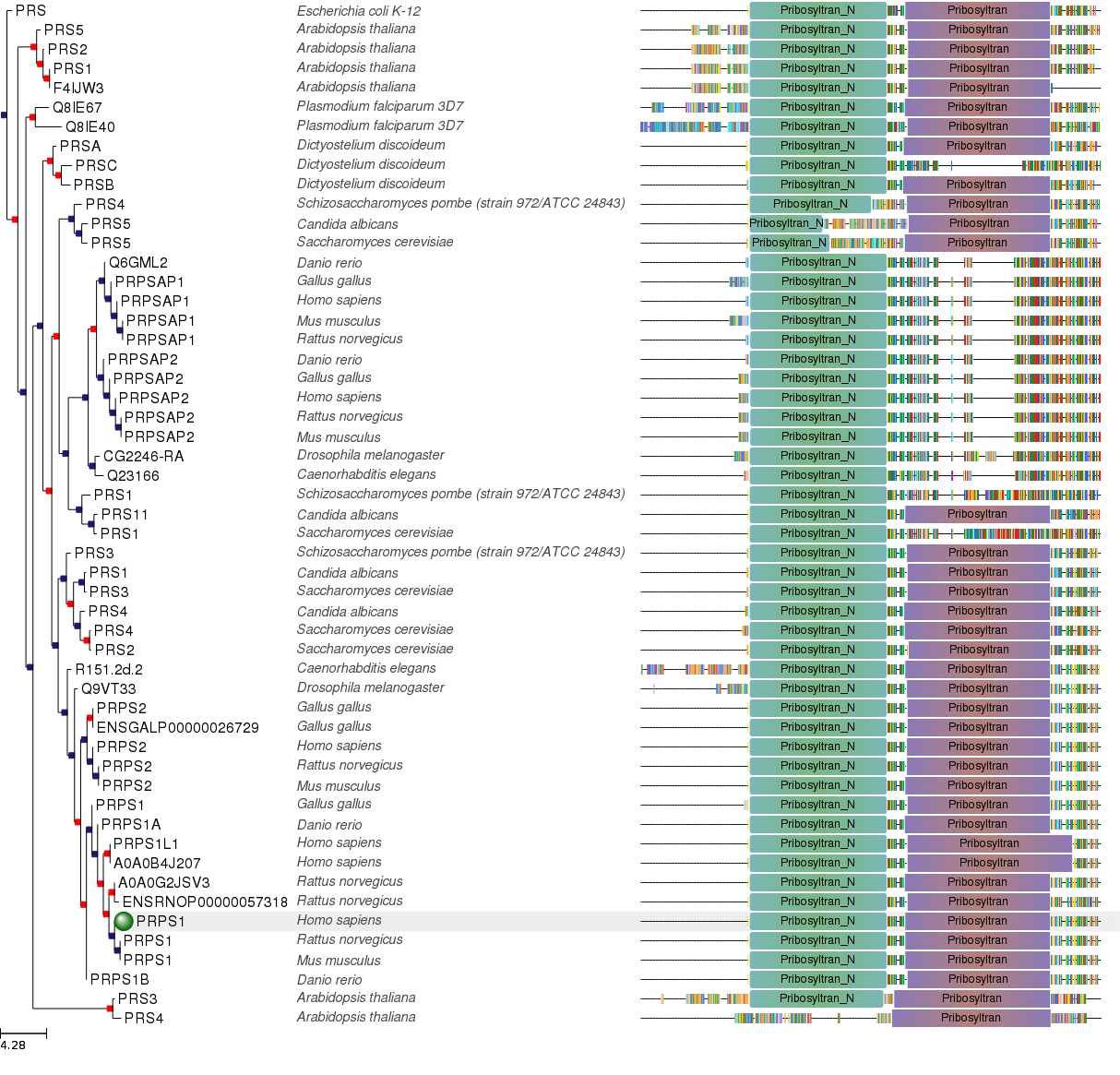
**

**Supplemental figure 1. Phylogenetic tree of PRPS and PRPS-binding proteins (PAP) across species**. Phylogenetic relationships of PRPS homologs across species were estimated using PhylomeDB (<http://phylomedb.org/> and [61]). Red indicates duplication events. Blue indicates speciation events. Green dot marks the target sequence, human PRPS1. Q9VT33 is the only Drosophila PRPS ortholog. PRPS-associated protein 1 and 2 (PRPPSAP1 and PRPPSAP2) are orthologues of human PAP39 and PAP41, respectively. Conserved domains of PRPS-family are shown in the right panel.
